# The Influence on Mortality of the Antiseptic Treatment in Wounds on the Head

**Published:** 1879-11

**Authors:** Herman Mynter


### THE INFLUENCE ON MORTALITY OF THE ANTISEPTIC TREATMENT IN WOUNDS ON THE HEAD.

TRANSLATED FROM SWEDISH BY HERMAN MYNTER, M. D.

Professor Estlander, in Helsingfors, has since 1860 treated 341 cases of lesions of the head, the lesions of the face not counted. The treatment, from 1860 to 1869, was according to old-established principles, but from 1870, Lister's antiseptic treatment has been used.

He divides his cases into three groups: 1, simple wounds; 2, wounds with denuded bone; 3, wounds with fracture of the skull, and lesions of the brain.

The mortality in the first group, simple wounds, scarcely shows any difference in the two periods; from 1860 to 1869, eighty-two cases, with three deaths; from 1870 to 1877, ninety-five cases, with three deaths.

In the second group, wounds with denuded bone, the difference in the mortality is quite evident, being in the first period seven deaths in thirty-seven cases, or 19 per cent.; in the second period one death in sixty-seven cases, or 1.5 per cent. By using antiseptic treatment, the author regards the prognosis as favorable as that of simple wounds.

The third group, especially wounds with fracture of the skull and lesions of the brain, shows the advantage of the antiseptic treatment, nine out of twelve, or 75 per cent., dying in the first period, two out of thirteen, or 15 per cent., in the second period. Cases that terminated fatally during the first twenty-four hours are not counted. The same improvement in the prognosis by antiseptic treatment is seen in other lesions. Of thirty-one cases of complicated fracture of the extremities he lost sixteen in the first period, and of ten cases of penetrating wounds of the knee-joint six, while in the second period, with antiseptic treatment, of sixty-six cases of complicated fractures but nine, and of twelve cases of penetrating wounds in the knee-joint, but two died.—*Nordiskt Medicinskt Archiv.*

